# Chronic Affections of the Joints

**Published:** 1828-10

**Authors:** 


### CHRONIC AFFECTIONS OF THE JOINTS.

*Cases of Chronic Affections of the Joints, treated at the* Royal Infirmary of Edinburgh.*

In the case of Thomas Sprunt, aged thirty-nine, who was admitted on the 5th of May, you had an example of caries and ulceration of the ankle-joint, of which the history is detailed in the following report:

"A little in front of the right malleolus externus, there is the orifice of a sinus which runs underneath the extensor tendons, and terminates by another opening a little below the inner malleolus: from both of these orifices the probe may be passed into the articulation of the astragalus and tibia, the articulating surfaces of which bones are felt rough; discharge copious, but healthy; the motions of the joint are very confined, and the surrounding parts much swelled and slightly inflamed. General health pretty good; pulse 100; tongue a little furred; slight sweating; bowels rather costive.

"Twenty-six years ago was affected apparently with necrosis of the tibia, which bone is evidently much enlarged. Nine years ago was first attacked with his present complaints, from which he recovered completely in five months, and continued perfectly well until within six months of the present date, when he again relapsed after exposure to cold and wet."

This was a case in which, considering all its circumstances, no reasonable prospect of relief could be held out from any milder means, and, the man having come prepared for the removal of his leg, it was amputated by the single flap operation, two or three days after his admission.

You saw that in this case I had some difficulty in securing the blood-vessels: the tibial arteries were cut across, just at the point where they spring from the popliteal, and were

* Ibid.

found firmly imbedded in the condensed cellular membrane, adhering to the bone, so that it was necessary to separate them with a scalpel from the posterior surface of the tibia, before I could surround the vessels with a ligature: the parts were obviously much altered in consequence of the pre-existence of inflammatory action in the course of the tibia, and the state of the bone itself was far from being satisfactory. It afforded a remarkable contrast to the state of the radius and ulna in Gardiner's forearm, which I had amputated only a few minutes before: there the bones possessed the sound and healthy tint with which every surgeon is familiar, and the blood oozed freely from their cut surfaces. In Sprunt's case, on the contrary, the tibia was dry, of a deadly whiteness, and not a particle of blood or of marrow oozing from its cut surface. This led me to fear that I had erred in amputating below the knee: the result, however, proved highly satisfactory; the stump healed kindly, although slowly, and the patient left the hospital full of gratitude, expressing his satisfaction at having exchanged his diseased leg for a wooden one.

This man's leg and ankle were exhibited to you immediately after the operation, and I have now again the pleasure of shewing you the bones in an advanced, although not in a complete, state of preparation: the bones of the tarsus, and particularly the astragalus, although somewhat softened, and in some points deprived of their cartilages, are, upon the whole, much less diseased than I expected to find them. The tibia appears to have been the seat of long standing and inveterate disease: this bone is enlarged throughout its whole length, but more particularly at its extremities; and, on laying it open by a longitudinal section, you observe its whole medullary cavity occupied by osseous deposition. This internal structure has been, in several places, the seat of caries, or perhaps I should rather say of internal abscesses: a small one is observable just at the point where the bone was cut across in removing the limb, another immediately below this point, a third towards the middle of the bone, and a fourth very large cavity is situated in the distal extremity of the tibia, immediately above its junction with the astragalus: this cavity, in the recent state, contained a quantity of purulent matter. It opens in front by a circular aperture immediately over the ankle-joint; and it was, I suspect, into this opening that the probe passed when I conceived it to be going into the cavity of the joint: on the posterior surface of the bone, nearly opposite to the seat of this abscess, you will find some adventitious deposition of ossific matter in a stalactitic form.

The fibula scarcely presents any thing worthy of notice : its lower extremity is divested of its articular cartilage, and apparently roughened ; but whether this is the effect of disease or of maceration, is not very easily determined.

During the present course, several instances of the Morbus Coxarius, or disease of the hip-joint, have fallen under your observation in various stages of its progress.

In the case of Mary Robertson, aged twelve, a patient of mine, you had an opportunity of seeing this affection in an incipient state, of which the following circumstances were symptomatic: "Complains of pain inside the left thigh and knee, much increased by exercise; the limb is slightly elongated, apparently from inclination of the pelvis to the same side; the buttock is flatter than the opposite one; the thigh is a little inclined forwards, and the fold between it and the buttock is very indistinctly marked. All the motions of the limb are much impaired, but chiefly extension and flexion, more particularly the latter; pulse 120; some sweating; tongue furred; skin at present cool; appetite good. Complaints are of three weeks' duration, and began without evident cause."

This little girl was greatly relieved by leeching, anodyne fomentation, and confinement to the horizontal posture; and, after having been about six weeks under treatment, she left the hospital on the 21st of June, apparently free from disease.

In the case of Elizabeth Orrock, aged seventeen, a patient on Dr. Hunter's side of the house, the disease was further advanced and better marked. This girl stated, "that about eight months ago she began to feel some pain and stiffness in right hip-joint, which has been gradually increasing till within a month, since which time it has been increasing more rapidly. Pressure over the trochanter, or the motion of the joint, causes great pain. There appears to be a slight curvature of the spine in the lower dorsal vertebræ towards the left side. The anterior superior spinous process of ilium appears somewhat higher on left side than on right. The right leg appears elongated for about one and a half inch; the toes are everted; there does not appear any swelling or redness about joint; the limb lies easiest in the extended position; has at times throbbing pain in joint; no shivering; catamenia natural; general health good in every other respect."

This case was treated, in the first instance, by the application of leeches, several moxas being subsequently applied contiguous to the joint; and, on the 13th instant, a blister was placed upon the groin and interior part of the thigh, by which means she has been partially relieved.

With reference to the two last-mentioned cases, I offered you some extended observations on the hip disease, which, from its frequent occurrence and dangerous nature, becomes one of much importance. I shewed you that the elongation of the limb, which is so marked a feature in the early stages of this affection, although it may be in some slight degree influenced by changes in the joint itself, by swelling of the cartilages, or effusion within the capsule, is in all cases, when it becomes so conspicuous as in Orrock's case, to be explained only by the oblique position which the pelvis assumes. From the tenderness of the affected joint, the patient is induced to throw the weight of his body upon the top of the sound thigh, while the affected limb is generally stretched out and thrown forwards, as a stay to prevent him from falling, and to assist him in progression. The pelvis is thus gradually, and almost imperceptibly, elevated on the sound side, while it drops proportionally on the diseased one; and this obliquity often continues to increase until the patient becomes bedridden, when we frequently find the limb shortened, and the position of the pelvis reversed. This appears to me to be in a great measure owing to a continued state of contraction in the psoas magnus muscle of the affected side, which, originating by different slips from the sides and transverse processes of the lumbar vertebræ, passes down to be inserted into the trochanter minor: its contraction, therefore, tends to approximate the transverse processes on the diseased side, and to give a lateral curvature to the lumbar portion of the spine. This is perhaps aided by the patient's lying for the most part on the sound side, resting upon the crest of the ilium, and thus pushing the pelvis towards the opposite side.

In more advanced stages of the disease, we have a shortening of the limb from circumstances less equivocal, and of which the explanation is abundantly obvious: such are the destruction of the ligaments, the absorption of the head of the bone, and consequent luxation of the joint; the absorption, in some instances, of the brim of the acetabulum, and in others of the bottom of this receptacle, with the consequent intrusion of the head of the bone into the cavity of the pelvis; of all which I shewed you examples.

Amongst other preparations illustrating the ravages of this disease, I shewed you an interesting, and perhaps an unique, specimen from Dr. Knox's collection, which gives at once a view of all the consequences above enumerated.

Here you saw, on the right side, the acetabulum obliterated, and in its site a rough irregular surface, with scarcely any vestige of an articular cavity: the head of the thigh-bone, on this side, divested of its usual globular form, its surface rough, and the limits between it and the cervix femoris very indistinctly marked. On the left side, the os innominatum is smaller than its opposite fellow; in the site of the acetabulum there is a complete breach or perforation in the bone, with some preternatural ossific deposit above it near the anterior and inferior spinous process of the ilium. The head and neck of the femur on this side have been completely absorbed as far down as the trochanter major: this process remains, and the intermediate portion of bone between it and the lesser trochanter is rough and quite irregular in its surface; below this point the femur is smooth, and shrunk in every dimension; and this wasting or atrophy extends to the other bones of the limb, the tibia and fibula being both shorter as well as smaller than their fellows of the opposite side. Of this singular preparation, I expect we will be furnished with a much more minute and perfect description in a work on the Pathology of the Bones, by Mr. BENJAMIN BELL, now in the press. The history of the subject from whom it was taken, a lad apparently about eighteen or twenty years of age, is unfortunately unknown. Dr. Knox has, however, very kindly offered me permission to take a sketch of this preparation, and I give it to you as a valuable and impressive memorandum of the nature and occasional consequences of this destructive disease.

In the treatment of the morbus coxarius, our object is to subdue the inflammatory action within the joint, and this we attempt, in the early and acute state of the disease, by the repeated application of leeches; and subsequently by the use of what are termed counter-irritants, blisters, moxas, caustic issues, and setons. This last class of remedies may perhaps be employed here with less equivocal effects, and with a better prospect of success, than in other joints more superficially covered, where it appears to me doubtful how far we can establish an inflammatory action on the surface without the risk of its extending to the interior. Whatever affords a rational prospect of relief ought to be employed with particular assiduity and perseverance in diseases of the hip-joint, because the removal of the limb, to which we often resort with success in the diseases of other joints, is here scarcely admissible, or at least its practice in this disease has not been encouraging. The excision of the head of the bone in some aggravated cases, where the articular apparatus has been destroyed and the joint dislocated, is, in my opinion, a much more promising operation; and I mentioned to you a very interesting case in which it was performed successfully by SCHMALZ, and which you will find detailed by HEDENUS of Dresden, in an excellent thesis, "De Femore in Cavitate Cotyloidea amputando."

